# Science Has to Take Responsibility. 10 Years World Health Summit—The Road to Better Health for All

**DOI:** 10.3389/fpubh.2018.00314

**Published:** 2018-10-14

**Authors:** Detlev Ganten, João Gabriel Silva, Fernando Regateiro, Ali Jafarian, Hélène Boisjoly, Antoine Flahault, Ben Canny, José Otávio Auler, Julian Kickbusch, Jörg Heldmann, Axel Pries, Michael J. Klag

**Affiliations:** ^1^World Health Summit, c/o Charité-Universitätsmedizin Berlin, Berlin, Germany; ^2^Centro Hospitalar e Universitário de Coimbra, Coimbra, Portugal; ^3^Tehran University of Medical Sciences, Tehran, Iran; ^4^Université de Montréal, Montreal, QC, Canada; ^5^University of Geneva, Geneva, Switzerland; ^6^University of Tasmania, Hobart, TAS, Australia; ^7^Faculdade de Medicina da Universidade de São Paulo, São Paulo, Brazil; ^8^Charité-Universitätsmedizin Berlin, Berlin, Germany; ^9^Johns Hopkins Bloomberg School of Public Health, Baltimore, MD, United States

**Keywords:** global health, agenda setting, health in all policies, sustainable development goals (SDG), interdisciplinary

## Summary

Science has to take responsibility! Our common future is already determined by research and by new technologies emerging from new knowledge, and this process continues to gain speed. We have to make sure that today's amazing developments are used to benefit all of humankind. This is what people expect from the progress. Health and wellbeing is what people are most concerned about. Health may be a human right but when it comes to health, the world is in a worrisome state despite all the great progress, which we have seen in the past years. Infectious diseases and pandemic preparedness remain on the world agenda but the chief causes of death are now shifting dramatically to non-communicable diseases. The burden of disease of e.g., obesity, diabetes, cardiovascular diseases, cancer, and mental health are increasing in all countries around the globe—rich and poor. In addition food and nutrition, pollution, climate change, political instability in many regions, economic crisis, migration, and flight are all contributing factors in major health issues. Meanwhile, the world's population continues to grow and to age. The global burden of disease remains, and is even on the rise in some areas. Equity remains a challenge. This may not be completely new but it is urgent for us to act. Climate is just one example that action is needed—now! We are not making good use of our scientific and technological possibilities and we are not living up to the expectations of the generations of our children and grandchildren.

Ever since the first World Health Summit on the occasion of the 300th anniversary of the Charité–Universitätsmedizin Berlin, we have been working toward one goal: improving health for everyone on the planet. We have to respond to the most important global health challenges, and must find fast and efficient ways to bring medical advances to the places where people are in need. But each of us can do very little alone. That's why from the beginning, the World Health Summit has brought together stakeholders and decision-makers from every sector, from all over the world. By combining forces from academia, the private sector, industry, civil society, and politics, we have been able to achieve change and improve health worldwide. And there are now some very promising leads on how we can do even more.

The “M8 Alliance” of 25 Academic Health Centers and Universities around the globe and the 130 Academies of Medicine and Sciences in the InterAcademy Partnership (IAP) provide a unique think tank for the World Health Summit program in academic freedom. We try to help setting the global health agenda including the G7/G20 Summits and stimulating the building up of global health structures, careers and programs in institutions and nations—and inviting politics, industry, and civil society to cooperate in a transparent way.

A milestone—and an encouraging sign—is the prominent position that health topics have assumed on the agendas of the G7 and G20 meetings. From the very beginning, the World Health Summit has enjoyed support from the highest level of politics, with ongoing high patronage from the Chancellor of Germany, the President of the French Republic and the President of the European Commission.

The Sustainable Development Goals (SDGs) and Agenda 2030 provide the framework for a holistic health approach in every area of policymaking. We do believe that such a holistic approach to health, is frequently discussed but is still largely neglected by too many people with responsibility and by too many institutions and urgently needs strengthening. In fact, the fragmentation of approaches, disciplines, particular interest groups and ideologies is obvious in science as well as in politics, private sector, and civil society. The challenge of the future is to cope with complexity. Global Health is one of the most complex issues—it would be a great example to develop a comprehensive holistic and successful model fitting for the great variety of good intentions and fragmented efforts.

It is encouraging to see important NGOs, private foundations and other key organizations playing an increasingly supportive and coordinated role in achieving the Sustainable Development Goals in tandem with the United Nations and the World Health Organization. We need efforts that are transdisciplinary, science-based, cross-sectoral, and concerted. They are vital to set the global health agenda for the years to come. Participants from all over the world bring different views, experiences, and priorities. They aren't only welcome—they're the very essence of the World Health Summit vision, mission and philosophy.

## The WHS—a strategic forum

The World Health Summit (WHS) was established in 2009 as an international, interdisciplinary conference in Berlin. From the start the goal was to find faster, more efficient and more equitable ways to advance health on a global scale. This could not be achieved through bringing together research and academia alone, many other stakeholders were required: politics, civil society, and the private sector. The WHS highlights the joint efforts in working toward one goal—improving health for everyone on the planet.

From the very beginning it was clear to us that science has to take more responsibility and that it was important to create a novel forum which had the *whole picture* as its guide.

From the very beginning, the WHS has enjoyed support from the highest levels of European government, with the consistent high patronage of the Chancellor of Germany, the President of the French Republic and the President of the European Commission. Several heads of state, leaders of international organizations and NGOs, CEOs of industry and leading members from academia have confirmed their participation in the 10th anniversary summit in 2018—10 years on, the World Health Summit has become the foremost international multisector strategic forum for global health. It has also generated regional conferences and expert meetings on specific topics around the globe.

From the beginning it was the hallmark of the WHS to include the best of science and all aspects of its translation into global health. The goal, however, was to go beyond medicine and to develop a truly holistic view of health encompassing all complex aspects of biology, medicine and the specific conditions we live in, our environment in the different parts of the world and importantly our behavior, lifestyles, social, and economic determinants as well as the various cultures around the world. This interface is clearly reflected at the 10th anniversary in 2018 in Berlin: on this occasion the World Health Summit is organized jointly and back to back with the “Grand Challenges Partners” including Grand Challenges Canada, the German Ministry of Education and Research, USAID, the Wellcome Trust and the Bill and Melinda Gates Foundation.

The WHS can look back on several major scientific and political milestones: in international scientific cooperation the start was the founding of the *M8 Alliance of Academic Health Centers, Universities and National Academies of Medicine and Sciences*—the Summit's academic think-tank from its very early days. This M8 Alliance importantly includes the InterAcademy Partnership IAP of all 130 National Academies of Medicine and Science around the world. The World Health Summit contributed to political agenda setting and the prominent position which health topics have assumed on the agendas of the G7 and G20 Summits since the Heiligendamm G8 Summit in 2007, strongly and independently also supported by the National Academies of Sciences and based on the increased commitment of several countries including Germany to global health. Issues that have been addressed at the WHS by leading international experts contribute to set the political health agenda and vice versa progress continues to be discussed on the programs of the WHS. New topics of global health are continuously added to the plenary sessions or smaller workshops of the WHS. With 2 major meetings per year and the expert meetings around the world now provide a reliable international platform for acute burning and long-term global health issues open to all stakeholders. Programs such as The Young Physicians Leaders (YPL) and the New Voices program have helped a young enthusiastic international global health community to flourish. The WHS continues to be a major stimulus to foster national and international science and education programs and sustainable structures for teaching and research of global health at universities. The last 10 years have shown a remarkable development of global health research, international programs and teaching in many institutions beyond the members of the M8 Alliance.

## The WHS—grounded in a scientific and holistic concept of global health

The Sustainable Development Goals (SDGs) of the United Nations and the Agenda 2030 with the World Health Organization (WHO) as the main player in global health, have provided the framework for and are based on a holistic approach to health in science and policy. The World Health Summit and the M8 Alliance share this holistic view and concept with the UN and WHO. This facilitates the participation of so many important organizations to play increasingly supportive roles in achieving those goals. This joint commitment and collaboration is necessary if we want to live up to the expectations and hopes of so many people around the world. With this great and productive diversity of actors, stakeholders, institutions and expertise in global health, it will be increasingly important and urgent to agree on such an inclusive and holistic view of health and on a common basis of facts, concepts and approaches. The “health in all policies” project of the InterAcademy Partnership (IAP) is an important part of this strategy.

The multidisciplinary and multisectoral approach in the political arena and of the WHS importantly is also driven by strong and new scientific arguments. Complex environmental factors, climate, pollution, preservation of nature, biodiversity and animal health have clearly been shown to be closely linked to and to have major effects on human health. This is the basis for the important concept of “One Health” and “Planetary Health.” Since the dawn of civilization there was no major distinction between diseases affecting humans and animals, and the interplay between their respective ecosystems was well accepted. Hippocrates' treatise “On Airs, Waters, and Places” explicitly recognized that the environment is inherently interlinked with health. Such thinking was revived in the nineteenth century by Rudolf Virchow, who proclaimed that “…between animal and human medicine there are no dividing lines–nor should there be,” which then heralded the necessity of the control of zoonoses such as the highly pathogenic avian influenza, Ebola, and rabies as well as the increasing challenge of antimicrobial resistance (AMR). Now we have precise knowledge of the exact transmission pathways from animals to man and back to animals. We have increasing insight into the vectors and hosts and we learn to control, treat and prevent infections.

This mandates interdisciplinary research closely linked to multi-sectoral policies and regulatory guidelines concerning poultry and livestock production, as well as marketing practices, food safety policies, and guidelines, legislation of the use of antibiotics in animals and their enforcement. At the international level, this has led to increasing collaboration between basic sciences and political agencies such as the World Health Organization (WHO), the Food and Agriculture Organization (FAO), and the Organization for Animal Health (OIE).

Basic research remains an increasingly important part in our efforts to work for a better future. Understanding complexity will remain the basis for unifying theories and holistic concepts. The Darwinian revolutionary hypothesis on the origin of species is such a strong unifying hypothesis. This has now become in resent years a new and very precise science that has been termed “Evolutionary Medicine” and “Evolutionary Global Health.” Such a development has become possible by the development of revolutionary new methods including genomics and genetics and the molecular analysis of the evolution of plants, animals, and man. These new insights have helped to develop a new theory of life. It provides new insights as to why human biology is the way it is, why people are frail and why they get sick and how to preseve better health rather than simply focus on treating diseases. A better understanding of the evolution of life on earth including health and diseases at the genomic and molecular level also provides a scientific rational for a holistic approach to health and disease integrating the complex interactions between our biology, the environment and our behavior. The gap e.g., between our evolutionary “old” biology and our modern, fast-changing, frequently man-made new environments such as cities and nutrition, helps to explain many diseases of civilization.

## The WHS—committed to SDGs and health in all policies

The SDGs respond to these complex interactions and provide a framework for a holistic answer to the ensueing challenges. Implementation of the SDGs started worldwide in 2016 in a process also referred to as “Localizing the SDGs.” All over the planet, individual people, universities, governments, institutions, and organizations of all kinds select “their goals” according to their expertise, capacities, means and knowledge. In each country, governments must translate the goals into their national legislation, develop a plan of action, establish budgets and openly and actively search for partners. The big challenge is to maintain the holistic view while encouraging diversity.

The World Health Summit's hallmark is this interdisciplinarity and the multistakeholder participation and interaction—this is one of the reasons the leaders of Ghana, Norway, and Germany have invited the WHO Director General to present at the WHS 2018 an action plan for the implementation of the SDGs including all relevant organizations and coordinated under the leadership of WHO. It reflects the importance of a forum which has the *whole picture* as its guide. “Good health and wellbeing” may be specifically mentioned just in SDG 3 but it is included in all 17 SDGs: from SDG 1 “no poverty,” SDG 2 “no hunger” to SDG 16 “peace, justice and strong institutions” to SDG 17 “partnerships for the goals.” Health is an extremely good entry point to the complexity of the SDGs because it is easily understandable as an important concern for life and death for the individual person and for society at large.

Improving global health requires input from many disciplines beyond and above medicine, biology, agriculture, nutrition, oceanography, and including the social and environmental sciences, humanities and engineering, allied health professions, all of which are essential for the implementation of health policies and programmes. This need for interdisciplinary action extends to “Health in All Policies,” an approach to public policies across sectors e.g. research, education, health, digitalization, economy, energy, foreign policy, security, and finances that systematically take into account the health implications of decisions of each of the government ministries and sectors to seek synergies and avoid harmful health impact to population and global health. Such initiatives have been asked for since the Alma-Ata Declaration in the 1970s but the results are far from being satisfactory in most countries. Climate is just one powerful example of how global trends (and actions to deal with them) affect health and require a “Health in All Policies” approach to be addressed effectively and avoid counteractive actions.

## The WHS—committed to diversity

Diversity of participants from all parts of the world, different cultures, professions, interest groups makes the meeting productive. The workshop “Respect and Dialogue” hosted by the M8 Alliance and InterAcademy Partnership IAP is an attempt to provide an intellectual basis for the SDGs and an example for exercise in constructive discussions in academic freedom. The “Health in all Policies” session is based on the view that health is a human right and a political choice. Other major topics of the World Health Summit 2018 such as “Pandemic Preparedness,” “Migration and Refugee Health,” “Health System Strengthening,” “Antimicrobial Resistance,” “The Digital Healthcare Revolution” all need to be integrated into the bigger holistic picture of global health. The issue of gender equity is addressed throughout the WHS.

The World Health Summit has become a unique forum to garner high-level political, civil society, academic and industry engagement in global health. It is further strengthened by the significant increase of support to global health by the German government and the relevance of Berlin as a global health hub. As stakeholders from areas other than health contribute to the World Health Summit, health actors need to ensure their participation at other fora such as the Munich Security Conference, or the Global Solutions Summit. By participating in these activities, steps will be made to ensure global health is represented in all political dialogues and ultimately in all policies.

Such transdisciplinary, science based, cross-sectoral concerted efforts are necessary to set the agenda for global health for the years to come. Participants from all over the world with their different views, experiences and priorities and interests are thus not only welcome—you are the essence of the World Health Summits vision, mission, and philosophy.

## Author contributions

All authors listed have made a substantial, direct and intellectual contribution to the work, and approved it for publication.

## Reading list

Beaglehole R, Yach D. Globalisation and the prevention and control of non-communicable disease: the neglected chronic diseases of adults. *Lancet* (2003) 362:903–8. doi: 10.1016/s0140-6736(03)14335-8
Bloom DE, Cafiero ET, Jané-Llopis E, Abrahams-Gessel S, Bloom LR, Fathima S, et al. *The Global Economic Burden of Non-communicable Diseases*. Geneva: World Economic Forum (2011). Available online at: http://apps.who.int/medicinedocs/documents/s18806en/s18806en.pdf (Accessed April 27, 2018).
Chopra M, Galbraith S, Darnton-Hill I. A global response to a global problem: the epidemic of overnutrition. *Bull World Health Organ*. (2002) 80:952–8.
Deloitte. *2017 Global Life Sciences Outlook* (2016). Available online at: https://www2.deloitte.com/content/dam/Deloitte/global/Documents/Life-Sciences-Health-Care/gx-lshc-2017-life-sciences-outlook.pdf (Accessed April 24, 2018).
Dye C, Acharya S. How can the sustainable development goals improve global health? A call for papers. *Bull World Health Org*. (2017) 95:666–A. doi: 10.2471/blt.17.202358
Ganten D, Nesse R. The evolution of evolutionary molecular medicine. *J Mol Med*. (2012) 90:467–70. doi: 10.1007/s00109-012-0903-2
Glover M, Buxton M, Guthrie S, Hanney S, Pollitt A, Grant J. Estimating the returns to UK publicly funded cancer-related research in terms of the net value of improved health outcomes. *BMC Med*. (2014) 12:99. doi: 10.1186/1741-7015-12-99
Glover M, Montague E, Pollitt A, Guthrie S, Hanney S, Buxton M, et al. Estimating the returns to United Kingdom publicly funded musculoskeletal disease research in terms of net value of improved health outcomes. *Health Res Pol Syst*. (2018) 16:1. doi: 10.1186/s12961-017-0276-7
Haines A. Health co-benefits of climate action. *Lancet Planetary Health* (2017) 1:e4–5. doi: 10.1016/s2542-5196(17)30003-7
Health in all policies (HiAP) framework for country action. *Health Promot Int*. (2014) 29 (Suppl. 1):I19–28. doi: 10.1093/heapro/dau035
*Ipsos Public Affairs: What Worries the World*. Available online at: https://www.ipsos.com/sites/default/files/2017-08/What_worries_the_world-July-2017.pdf (Accessed April 24, 2018).
Jacobs B, Ir P, Bigdeli M, Annear PL, Damme WV. Addressing access barriers to health services: an analytical framework for selecting appropriate interventions in low-income Asian countries. *Health Pol Plan*. (2011) 27:288–300. doi: 10.1093/heapol/czr038
M8 Alliance. *M8 Alliance Declaration. World Health Summit 2017. Health is a Political Choice* (2017). Available online at: https://d1wjxwc5zmlmv4.cloudfront.net/fileadmin/user_upload/downloads/2017/WHS_Berlin/Data/M8_Alliance_Declaration_2017_Berlin.pdf (Accessed April 24, 2018).
Sobocki P, Lekander I, Berwick S, Olesen J, Jönsson B. Resource allocation to brain research in Europe (RABRE). *Eur J Neurosci*. (2006) 24:2691–3. doi: 10.1111/j.1460-9568.2006.05116.x
Steyn K, Damasceno A. Lifestyle and related risk factors for chronic diseases. In: Jamison DT, editor. *Disease and mortality in Sub-Saharan Africa*. Washington, DC: World Bank (2006). Available online at: https://www.ncbi.nlm.nih.gov/books/NBK2290/ (Accessed April 24, 2018).
The Lancet. Life, death, and disability in 2016. *Lancet* (2017a) 390:1083. doi: 10.1016/S0140-6736(17)32465-0
The Lancet. *Climate Change and Health*. (2017b). Available online at: http://www.thelancet.com/infographics/climate-and-health (Accessed April 24, 2018).
United Nations. *2017 Revision of World Population Prospects*. (2017). Available online at: https://esa.un.org/unpd/wpp/Publications/Files/WPP2017_KeyFindings.pdf (Accessed April 24, 2018).
United Nations. *The World at Six Billion*. Department of Economic and Social Affairs (1999). Available online at: http://www.un.org/esa/population/publications/sixbillion/sixbillion.htm (Accessed April 24, 2018).
WHO/World Bank Group. *Tracking Universal Health Coverage: 2017 Global Monitoring Report* (2017). Available online at: http://www.who.int/healthinfo/universal_health_coverage/report/2017/en/ (Accessed April 24, 2018).
WHO. *Noncommunicable Diseases*. (2017). Available online at: http://www.who.int/en/news-room/fact-sheets/detail/noncommunicable-diseases (Accessed April 24, 2018).
Wooding S. *Project Retrosight: Understanding the Returns from Cardiovascular and Stroke Research: The Policy Report*. RAND Corporation (2011). Available online at: https://www.rand.org/pubs/monographs/MG1079.html (Accessed April 24, 2018).

### Conflict of interest statement

The authors declare that the research was conducted in the absence of any commercial or financial relationships that could be construed as a potential conflict of interest.

